# Preoperative estimation of breast resection weight in patients undergoing inferior pedicle reduction mammoplasty: the Bilgen formula

**DOI:** 10.3906/sag-1905-7

**Published:** 2020-06-23

**Authors:** Fatma BİLGEN, Alper URAL, Mehmet BEKERECİOĞLU

**Affiliations:** 1 Plastic, Reconstructive, and Aesthetic Surgery, School of Medicine, Kahramanmaraş Sütçü İmam University, Kahramanmaraş Turkey

**Keywords:** Mammaplasty, breast, hypertrophy, resection weight, estimation

## Abstract

**Background/aim:**

Symptomatic breast hypertrophy has a significant impact on the quality of life of women. The amount of tissue to be excised may be preoperatively estimated by an experienced surgeon. However, this remains a subjective assessment. Accurate quantification of the amount of breast tissue to be resected in the preoperative period will be a guide for both patient information and the surgeon during the operation. The aim of this study is to develop a new method based on simple measurements that can accurately estimate the resection weight in the preoperative period in a wide range of patients undergoing reduction mammoplasty.

**Materials and methods:**

The study was carried out between December 2016 and September 2018. With the determined drawing and measurement methods, a triangle was obtained by measuring the distances among the sternal notch (A) - right nipple areola midpoint (B), sternal notch (A) - left nipple areola midpoint (C) and both internipple areola (B-C). The height of this triangle (h) was found by measuring the distance between the sternal notch and the midpoint of both nipple areola levels. The amount of breast tissue to be resected for each breast was calculated by multiplying the distance between the sternal notch–nipple areola and the height of the large triangle. The formula may be expressed as AB × h for the right breast and AC × h for left breast.

**Results:**

When the t values ​​and significance levels of the beta coefficients of the independent variables were examined, the preoperative values ​​were determined to be in accordance with the actual values ​​after surgery (P < 0.05). The values ​​calculated before were calculated as the percentage of the actual values (91%). In other words, the R2 value showed that the calculated values were compatible with the actual values (R2 = 0.910).

**Conclusions:**

With the formula described herein, one may accurately estimate the amount of tissue to be resected in a wide range of patients undergoing reduction mammoplasty whose sternal notch–nipple distances are between 28–42 cm. Additionally, because measurements for each breast are performed separately, breast asymmetry does not affect the results. In conclusion, the formula we devised is simple, applicable, and has a high accuracy rate.

## 1. Introduction

Symptomatic breast hypertrophy has a significant impact on the quality of life of women. Reduction mammaplasty aims to reduce the symptoms of macromastia and provide the aesthetic appearance of a large ptotic breast. It has been shown that many symptoms such as neck and back pain, shoulder groove, intertrigo and posture disorders have disappeared, and quality of life, self–esteem and even pulmonary function have improved in patients who underwent reduction mammoplasty. Given all these reasons, it has become one of the most commonly performed surgeries by plastic surgeons in recent years [1–3].

The weight of the breast tissue excised in reduction mammoplasty may vary from a few hundred grams to 3 kg depending on the original breast size. Reduction mammoplasty techniques are described as standard, and the technique to be administered is preferred based on the size of the breast and the experience of the surgeon. The amount of tissue to be excised may be preoperatively estimated by an experienced surgeon. However, this remains a subjective assessment. Accurate quantification of the amount of breast tissue to be resected in the preoperative period will be a guide for both patient information and the surgeon during the operation [4,5].

Appropriate preoperative measurements will eliminate such problems. Several studies have been carried out to determine the amount of resection, and various formulae have been proposed [6,7]. However, widespread use of these formulae could not be achieved due to their complexity, difficulty of applicability, or the lack of sensitivity in measuring the amount of tissue resected at the end points.

The aim of this study is to develop a new method based on simple measurements that can accurately estimate the resection weight in the preoperative period in a wide range of patients undergoing reduction mammoplasty.

## 2. Materials and methods 

The study was conducted with Ethics Committee approval. It was planned as a prospective study, and 68 patients aged between 25–65 years (136 breasts) who underwent bilateral reduction mammoplasty (inferior pedicle) for symptomatic breast hypertrophy between December 2016 and September 2018 were included in the study.

The age, height, weight, and body mass index (BMI) of all patients were recorded before the operation. Following the determined drawing and measurement methods, a triangle was obtained by measuring the distances among the sternal notch (A)–right nipple areola midpoint (B), sternal notch (A)–left nipple areola midpoint (C), and both inter nipple areola (B-C).

The height of this triangle (h) was found by measuring the distance between the sternal notch and the midpoint of both nipple areola levels. Afterwards, during the preoperative marking procedures, bilateral new nipple areola points were marked on the level of the inframammary fold.

**Figure 1:**
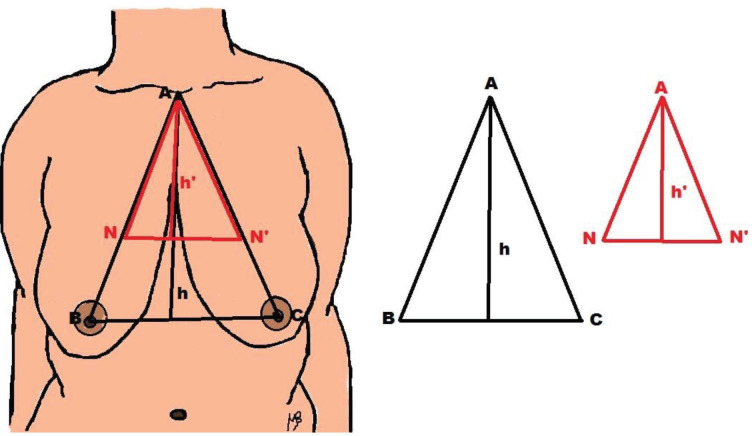
Drawings of triangles.

A new triangle was obtained by measuring the distances among the sternal notch (A)–right side new nipple (N), sternal notch (A)–left side new nipple (N’), and the new internipple areolar distance. The distance between the base of the triangle and the sternal notch (A) was accepted as the height of the small triangle (h’) (Figure 1).

The amount of breast tissue to be resected for each breast was calculated by multiplying the distance between the sternal notch–nipple areola and the height of the large triangle. The formula may be expressed as AB × h for the right breast and AC × h for the left breast. While measuring the distances from the nipple areolar points, the nipples’ exact midpoints were marked delicately. If the measurements with decimal numbers were 0.4 or smaller, they were rounded to the lower integer. If the decimal numbers were 0.5 or higher, then the measurements were received as one higher integer.

In patients whose distances between the sternal notch–nipple areola were shorter than 28 cm or longer than 42 cm, as the difference between the preoperative and postoperative measurements were higher, a new formulation was developed for these patients by including the small triangle in the measurements.

Based on the small triangle obtained in the preoperative measurements, the formulae AN × h’ for the right side and AN’ × h’ for the left side were developed. It was observed that, if we added the calculations of the small triangle to the result of the first formulae’s calculations (right breast: [AB × h + (AN × h’)], left breast: [AC × h + (AN’ × h’)]) in the patients whose sternal notch–nipple areola distance was above 42 and subtract the calculations of the small triangle from the results of the first formulae’s calculations (right breast: [AB × h - (AN × h’)], left breast: [AC × h - (AN’ × h’)]) in patients whose sternal notch–nipple areola distance was below 28 cm, the estimated and the actual results were closer to each other. Consideration should be paid to evaluation of patient groups in cases of more extreme endpoints.

The drawings and measurements of all patients were performed by the first author, while the operations were carried out by 3 surgeons. After the calculations, all patients were operated on under general anesthesia. Each patient underwent breast reduction with the same technique, inferior pedicle and inverted T scar pattern. The amount of tissue resected from each breast was weighed separately and recorded.

### 2.1. Statistics

All obtained parameters were analyzed by using the SPSS for Windows (SPSS 10.1.3., SPSS Inc., Chicago, IL, USA) software. The prescribed breast tissue was resected in the operating theatre and weighed. For the statistical analyses, Pearson’s correlation coefficient and paired samples t test were utilized. The rate of compatibility between the resected breast tissue amounts and the estimated breast tissue amounts based on the formulae was evaluated by using linear regression analysis. In linear regression analysis, the amount resected in the surgery was the dependent variable, while the amount calculated before the surgery with the specified formula was determined as the independent variable. A P value of < 0.05 was considered statistically significant.

## 3. Results 

Sixty-eight patients aged between 25 and 62 years (mean: 44 years old) were enrolled in the study. The mean BMI of the patients was found to be 32.4, and the mean amount of resected breast tissue was 1112.43 g per breast. The predicted weight of the resection amount based on the formula was 1121.13 g per breast. The mean sternal notch–nipple areola distance was 35 cm (Table 1).

**Table 1:** Patient demographics.

	Mean ± SS	Range
Age (year)	44 ± 9.68	21–61
Body mass index (kg/m2)	34.2 ± 2.34	28–40.4
Sternal notch (A)-right nipple areole (B)	35.6 ± 4.10	27–45
Sternal notch (A)-right nipple areole (C)	35.7 ± 4.02	27–45
Total weight of resection (g per side)	1105.35 ± 293.69 (left)1119.52 ± 300.46 (right)	290–2280350–2460
Predicted weight of resection amount based on formula	1119.44 ± 241.12 (left) 1122.83 ± 243.10 (right)	621–1890 621–1848

**Figure 2:**
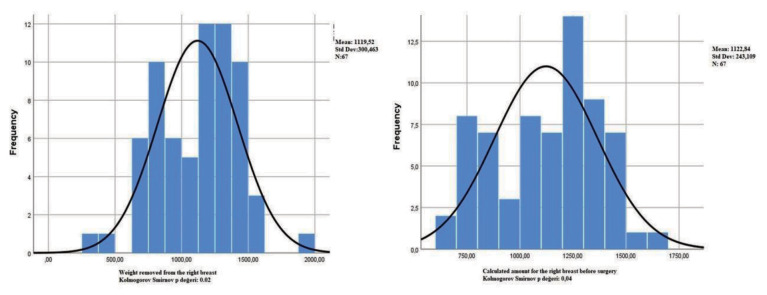
Right g: weight removed from the right breast; XA_×_h: calculated amount for the right breast before surgery (P value for the distribution of errors in Kolmogorov-Smirnov normality test: 0.200).

**Figure 3:**
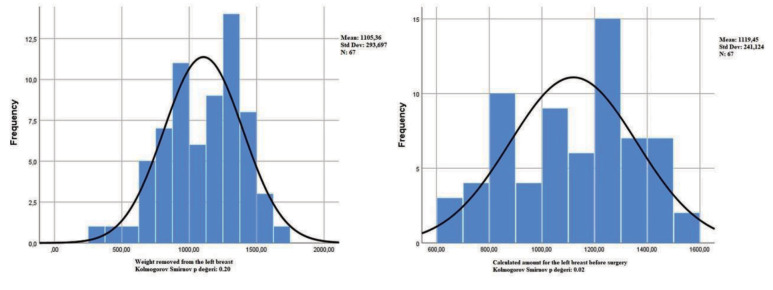
Left g: weight removed from the left breast; XB_×_h: calculated amount for the left breast before surgery (P value for the distribution of errors in Kolmogorov-Smirnov normality test: 0.171).

Parametric methods were used since the data satisfied the assumption of normal distribution (P < 0.01**)**. For normality, the Kolmogorov-Smirnov test statistics were as P: 0.20 for the distribution of errors in the right breast (P: 0.02 for the amount resected from the right breast and P: 0.04 for the amounts calculated for the right breast before the operation) and as P: 0.171 for the distribution of errors in the left breast (P: 0.20 for the amount resected from the left breast and P: 0.02 for the amounts calculated for the left breast before the operation) (Figures 2, 3).

Pearson’s correlation analysis was performed to determine the linear relationship between the weight of the resected tissue from the right breast and the estimated weight of the breast tissue to be resected from the right breast before the operation. According to the results, a statistically significant and strong positive correlation was found between the values ​​(P < 0.05; r = 0.958) (Table 2).

**Table 2:** The relationship between the weight of the mass removed from the right breast and the calculated weight of the mass in the right breast before surgery.

Variable	Right_g	XA_×_h
Right_g	1	
XA_×_h	0.958	1

Right g: removed weight from the right breast XA_×_h: calculated resection amount of right breast before surgery

Pearson’s correlation analysis was performed to determine the linear relationship between the weight of the resected tissue from the left breast and the estimated weight of the breast tissue to be resected from the left breast before the operation. According to the results, a statistically significant and strong positive correlation was found between the values ​​(P < 0.05; r = 0.955) (Table 3).

**Table 3:** Analysis of the relationship between the removed tissue weights from the left breast and the estimated tissue weights depending on calculation in the left breast before surgery.

Variable	Left_g	XA_×_h
Left_g	1	
XB_×_h	0.955	1

Left g: removed weight from the left breast XA_×_h: calculated resection amount of left breast before surgery

The paired samples t test was used to determine the difference between the values ​​calculated from the right and left breasts and the actual weights of the mass removed from the right and left breasts. H01: There is no significant difference between the values calculated for the right breast before the operation and the actual weight of the mass resected from the right breast after the operation. H02: There is no significant difference between the values calculated for the left breast before the operation and the actual weight of the mass resected from the left breast after the operation.

According to the test results, the hypothesis was not rejected with 95% confidence that there was no statistically significant difference between the preoperative predicted resection weight values ​​of both the right breast and the left breast and the actual weight values ​​of the resection amounts that were removed as a result of the operation. Accordingly, the difference between the calculated values ​​and the actual values ​​was not statistically significant (P > 0.05) (Table 4).

**Table 4:** The paired sample t test to determine the difference between the calculated values ​​from the right and left breasts before surgery and the actual weight of the mass removed from the right and left breasts after the operation.

Variables	Measurements	N	X̄ ± SS	t	P
Right breast	Resection values	67	1119.52 ± 300.46	–0.279	0.781
	Calculated values	67	1122.83 ± 243.10		
Left breast	Resection values	67	1105.35 ± 9.89	–1.202	0.234
	Calculated values	67	1119.44 ± 241.12		

Both the independent and dependent variables were continuous, and their distributions were normal. There was a linear relationship between the independent and dependent variables. A regression analysis was conducted, and the model was found significant. The errors of the model had a normal distribution. There was no problem of autocorrelation in the model. If the DW test statistic is between 1.5–2.5, this means there is no problem of autocorrelation.

A simple linear regression analysis was performed to explain the effect of the preoperatively calculated values ​​on the actual values ​​obtained after the study. When the significance level corresponding to the F value was examined, it was seen that the model that was established was statistically significant (F = 721.476; P < 0.05). When the t values ​​and significance levels of the beta coefficients of the independent variable were examined, the preoperative values ​​were determined to be in accordance with the actual values ​​after surgery (P < 0.05). The values ​​calculated before were calculated as the percentage of the actual values ​​(92%). In other words, R2 showed the compliance of the calculated values ​​with the actual values ​​(adjusted R2 = 0.916) (Table 5). The adjusted R2 value of 0.916 showed that 91.6% of the weight resected from the right breast could be explained by the amount calculated before the operation for the right breast.

**Table 5:** Regression analysis results of the calculated tissue amount values in the right breast before the operation, for the actual weight of the removed tissue from the right breast after surgery.

Dependentvariable	Independentvariable	ß	T	P	F	Model(P)	Adjusted R2	DW
Right_g	Constant	–209.62	–4.142	0,000	721.476	0.000	0.916	2.017
	XA_×_h	1.184	26.860	0.000				

Normally, there are multiple independent variables that affect the dependent variable. To reduce the complexity of the model and establish comprehensible, interpretable models, variables that do not have an effect on the targeted variable, or have a low or negligible effect are excluded from the study. For this reason, to eliminate the independent variables that are unnecessarily added, the adjusted R2 was used instead of the R2 which is known as a goodness of fit index. 

A simple linear regression analysis was performed to explain the effect of the preoperatively calculated values ​​on the actual values ​​obtained after the study. When the significance level corresponding to the F value was examined, it was seen that the model that established was statistically significant (F = 666.411; P < 0.05). When the t values ​​and significance levels of the beta coefficients of the independent variables were examined, the preoperative values ​​were determined to be in accordance with the actual values ​​after surgery (P < 0.05). The values ​​calculated before were calculated as the percentage of the actual values ​​(91%). In other words, the R2 value showed that the calculated values were compatible with the actual values (R2 = 0.910) (Table 6). The adjusted R2 value of 0.910 showed that 91% of the weight resected from the left breast could be explained by the amount calculated before the operation on the left breast.

**Table 6:** Regression analysis results of the calculated tissue amount values in the left breast before the operation, for the actual weight of the removed tissue from the left breast after surgery.

Dependentvariable	Independentvariable	ß	T	P	F	Model(P)	Adjusted R2	DW
Left_g	Constant	–196.16	–3.805	0.000	666.411	0.000	0.910	1.878
	XB_×_h	1.163	25.815	0.000				

It was shown that the formula used in the statistical analyses was successful in determining the amount of tissue removed, and it could be used in a wide range of patients whose sternal notch–nipple areola distance was between 28–42 cm. Although statistically significant results were obtained in all patients, according to the observations of the authors, the discrepancy between the preoperative values and the postoperative actual values was increasing in the patients whose sternal notch–nipple areola distance was below 28 cm or above 42 cm in comparison to the other patients.

## 4. Discussion

Several studies have shown significant improvements in symptoms and quality of life in patients with symptomatic breast hypertrophy following reduction mammaplasty [5,6]. In the study by Schnur et al., the majority of patients who underwent reduction mammaplasty stated that they required operation for symptomatic relief [7].

However, determining the tissue weight to be resected prior to reduction mammoplasty has become an important problem. As experienced surgeons can predict the amount of tissue to be resected in large breasts where the precise weight is not crucial, it is often difficult to predict in border macromastias. Preoperative determination of the amount of tissue to be resected will be helpful for surgeons during the operation. Additionally, insurers will have clear information about the coverage before the operation [1,3,6,7].

There are many equations based on anthropometric measurements and expensive scientific techniques such as the water displacement method (Archimedes), biometric analysis with stereometric cameras, various plastic cup-like devices and 3-dimensional imaging to estimate the weight of the breast tissue to be removed. However, application of the methods described above has not received widespread acceptance in usage because of their limitations such as being difficult or complicated or insufficiency in determining breast sizes at the end points [8–13].

Sommer et al. retrospectively examined 263 patients who underwent reduction mammaplasty, and they researched the association between sternal notch–nipple distance and resection weight. Although the generally applied formula was successful in predicting the resected tissue weight in large breasts, the accuracy rate for resections of less than 600 g was calculated as 50%. Additionally, it will not be possible to make accurate estimations with a single measurement due to the varying resection amounts in patients with breast asymmetry [14].

Descamps et al. analyzed 214 patients who underwent reduction mammaplasty in South Africa and aimed to determine the resection weight by a single formula. The correlation test between the sternal notch–nipple, inframammary fold-nipple distance, and BMI showed a significant correlation with the resection amount, but the formula created by sternal notch–nipple, inframammary fold-nipple distances could not predict the resection weight. It was also shown that reliability decreased in mild resection quantities and by only using vertical plan measurements as the independent variables [15].

On the other hand, Kocak et al. formulated the sternal notch–nipple distance by evaluating the vertical and horizontal measurements of the breast surface. They found that the parameter obtained by multiplying the vertical and horizontal measurements had a relatively high correlation coefficient (r = 0.95) with the resection weight of the breast tissue. They suggested that the sternal notch–nipple distance may be affected by the length of the rib cage, and only the formulae based on breast measurements provide more accurate results [16].

Regnault and Daniel developed a formula for estimating the resection weight required to achieve the desired bra size. The formula was based on the measurements of the chest circumference taken on the level of the nipple and from the level of the axillary region under the armpits. Although this method was useful for preoperative planning and estimation of cup size, it contributed little to predicting the required resection weights by the reason that its purpose was to calculate the amount of resection required to achieve the desired bra size. Additionally, it is not possible to estimate different weights from each breast in these cases with significant breast asymmetry [17].

Appel et al. examined the relationship of resection weight among sternal notch–nipple distance, inframammary fold to nipple distance and BMI in 349 reduction mammaplasty patients. By inclusion of all 3 parameters, a formula was obtained with a high degree of accuracy to obtain the estimated resection weight, but in the prospective evaluations, the formula was not introduced [18].

This study was prospectively performed on 68 patients, with 2 basic anthropometric measurements to define a simple formula that calculates the resection weight with an accuracy of over 90%. The measurements used here consisted of multiplying each lateral leg of the triangle obtained by combining sternal notch–nipple distances with the height of the triangle. The formula can be easily reproduced and calculated without the need for any tools. However, in measurements under 28 cm and over 42 cm, the difference between the resected amount of tissue and the amount calculated by the formula, and additional formulae are required. Additionally, as in other formulae used to estimate resection amounts, BMI is an important parameter.

Furthermore, thanks to the formula described herein, one can accurately estimate the amount of tissue to be resected in a wide range of patients undergoing reduction mammoplasty whose sternal notch–nipple distances are between 28–42 cm. Additionally, because each breast’s measurements are performed separately, breast asymmetry does not affect the results.

In conclusion, the formula we devised is simple, applicable, and has a high accuracy rate in patients with a BMI greater than 30. We believe that it can guide both patients and surgeons.

## Acknowledgements

All of the authors declare that they have all participated in the design, execution, and analysis of the paper, and they have approved the final version. Additionally, there are no conflicts of interest in connection with this paper and the material described is not under publication or consideration for publication elsewhere.
